# Genetic Characterization and Biofilm-Forming Capacity of Bacterial Population Isolated from Conjunctival Samples

**DOI:** 10.3390/antibiotics15030300

**Published:** 2026-03-15

**Authors:** Adela Voinescu, Silvia-Ioana Musuroi, Monica Licker, Delia Muntean, Florin-George Horhat, Luminita Mirela Baditoiu, Oana Izmendi, Andrei Cosnita, Mihnea Munteanu, Mihai Poenaru-Sava, Valentin Ordodi, Petrinela Ceachir, Tudor Rareș Olariu, Corina Musuroi

**Affiliations:** 1 Doctoral School, “Victor Babes” University of Medicine and Pharmacy, 300041 Timisoara, Romania; adela.voinescu@umft.ro (A.V.); silvia.musuroi@umft.ro (S.-I.M.); oana.izmendi@umft.ro (O.I.); petrinela.daliu@umft.ro (P.C.); 2 Multidisciplinary Research Center on Antimicrobial Resistance (MULTI-REZ), “Victor Babes” University of Medicine and Pharmacy, Eftimie Murgu Square 2, 300041 Timisoara, Romania; horhat.florin@umft.ro (F.-G.H.); baditoiu.luminita@umft.ro (L.M.B.); corina.musuroi@umft.ro (C.M.); 3 Microbiology Department, “Victor Babes” University of Medicine and Pharmacy, Eftimie Murgu Square 2, 300041 Timisoara, Romania; 4 Discipline of Clinical Skills, Department I Nursing, “Victor Babes” University of Medicine and Pharmacy, 300041 Timisoara, Romania; 5 Microbiology Laboratory, “Pius Brinzeu” County Clinical Emergency Hospital, 300723 Timisoara, Romania; 6 Clinical Laboratory, “Louis Turcanu” Emergency Hospital for Children, 300011 Timisoara, Romania; 7 Epidemiology Department, “Victor Babes” University of Medicine and Pharmacy, 300041 Timisoara, Romania; 8 Department IX, Surgery and Ophthalmology, “Victor Babes” University of Medicine and Pharmacy, 300002 Timisoara, Romania; cosnita.dan@umft.ro (A.C.); mihnea.munteanu@umft.ro (M.M.); mihai.poenaru-sava@umft.ro (M.P.-S.); 9 Center of Immuno-Physiology and Biotechnologies, Department of Functional Sciences, “Victor Babes” University of Medicine and Pharmacy, 300173 Timisoara, Romania; valentin.ordodi@umft.ro; 10 Research Center for Gene and Cellular Therapies in the Treatment of Cancer—OncoGen, “Pius Brinzeu” County Clinical Emergency Hospital, 300723 Timisoara, Romania; 11 Discipline of Parasitology, Department of Infectious Diseases, “Victor Babes” University of Medicine and Pharmacy, 300041 Timisoara, Romania; rolariu@umft.ro; 12 Clinical Laboratory, Municipal Clinical Emergency Hospital, 300254 Timisoara, Romania; 13 Center for Diagnosis and Study of Parasitic Diseases, Department of Infectious Disease, “Victor Babes” University of Medicine and Pharmacy, 300041 Timisoara, Romania; 14 Patogen Preventia, 300124 Timisoara, Romania

**Keywords:** bacterial conjunctivitis, phenotypic, genotypic, biofilm, *Staphylococcus aureus*, *Staphylococcus epidermidis*

## Abstract

Background/Objectives: Bacterial conjunctivitis is a common ocular infection requiring prompt treatment, particularly in vulnerable patients, and may influence perioperative outcomes. This study aimed to characterize conjunctival bacterial isolates phenotypically and genotypically, to evaluate their biofilm-forming capacity, and to investigate the relationship between resistance gene carriage, resistance phenotypes, and biofilm-associated antimicrobial resistance (AMR). Methods: A prospective, single-center, cross-sectional study was conducted on bacterial isolates from conjunctival samples of patients examined in an ophthalmology department. Antimicrobial susceptibility testing (AST) was performed to determine the minimum inhibitory concentrations (MICs). Resistance genes were detected by quantitative PCR. Biofilm-forming capacity was assessed using the microtiter plate assay, and biofilm susceptibility to amikacin (AK) and levofloxacin (LEV) was evaluated using a biofilm susceptibility assay. Results: A total of 78 isolates were analyzed; Gram-positive cocci prevailed (GPC, 84.6%), being significantly more frequent than Gram-negative bacilli (GNB, *p* < 0.001). Among GPC, 65.2% were multidrug-resistant, with *Staphylococcus epidermidis* emerging as the most frequent species (*p* < 0.001). Resistance gene carriage was detected in 33.3% of GNB. Strong biofilm formation was observed in 22.7% of GPC versus 58.3% of GNB. It should be noted that the relatively small number of GNB may limit the statistical robustness of comparisons between Gram-positive and Gram-negative groups. A statistically significant association between resistance genes and biofilm capacity was found only in *Staphylococcus aureus* (*p* = 0.027). Biofilm-embedded bacteria showed increased antimicrobial tolerance, particularly for AK in *S. aureus* and for both AK and LEV in *S. epidermidis* (*p* < 0.001). Conclusions: The prevalence of multidrug-resistant conjunctival isolates and their biofilm-forming capacity highlights the clinical importance of biofilm-related resistance and support integrating AMR profiling with biofilm assessment to optimize empirical therapy in bacterial conjunctivitis.

## 1. Introduction

Bacterial conjunctivitis represents the most common ocular infection, affecting both pediatric and adult populations, and requires prompt and effective intervention. The clinical importance of this condition is underscored by its potential for severe evolution, as the infection may lead to significant visual impairment or even vision loss, particularly in the presence of impaired anti-infective defense mechanisms, such as those encountered in neonates, burn patients, polytraumatized individuals, or critically ill patients admitted to intensive care units [1].

Recent literature data have demonstrated that the ocular surface of healthy individuals is colonized by a specific, relatively stable microbiome, characterized by the presence of an omnipresent core microbial community [2,3].

Among the bacterial genera most frequently identified as part of this core are *Pseudomonas*, *Propionibacterium*, *Corynebacterium*, *Acinetobacter*, *Staphylococcus*, *Sphingomonas*, and *Streptococcus*, with interindividual variations in their relative proportions caused by personal habits, systemic diseases, or antibiotic usage, thereby conferring microbiological uniqueness to each individual [2,4,5].

Under physiological conditions, the microbial community of the ocular surface exists in a dynamic equilibrium with host tissues, playing a critical role in maintaining local homeostasis and protecting against colonization by pathogenic strains. Disruption of this balance, whether due to local or systemic factors or to immunodeficiency, promotes the proliferation of opportunistic microorganisms within the resident microbiome [5]. In the context of compromised immune defenses, ocular infections may be caused not only by adjacent commensal bacteria (such as *Staphylococcus epidermidis*, a commensal of the lacrimal canaliculus), but also by pathogens originating from distant anatomical sites, including the respiratory or intestinal tract (e.g., *Pseudomonas aeruginosa, Acinetobacter baumannii*) [6,7].

The clinical relevance of these microorganisms is further enhanced by their behavior within microbial communities, particularly their ability to form biofilms, and to harbor antibiotic resistance genes, often acquired in other infectious contexts of the host. Biofilm formation is associated with bacterial persistence, chronicity of infection, and reduced therapeutic efficacy [8]. These aspects are of particular importance in ophthalmology, especially in the preoperative and postoperative setting, where the presence of resistant or biofilm-forming strains on the ocular surface may facilitate the development of iatrogenic infections [9]. Postoperative endophthalmitis following cataract extraction is primarily caused by Gram-positive (GP) organisms, particularly *S. epidermidis*, and cases involving antibiotic-resistant strains have been reported even in the context of intracameral antibiotic prophylaxis [10,11].

Accordingly, biofilm formation on glaucoma drainage devices has been associated with chronic, treatment-resistant endophthalmitis, requiring complex therapeutic approaches and, in certain cases, explanation of the drainage device [12]. Additionally, the literature evidence indicates that microorganisms are capable of adhering to and forming biofilms on the surface of intraocular lenses, with biofilm involvement in the pathogenesis of persistent or recurrent postoperative infections, including chronic endophthalmitis [7,9]. These mechanisms are also relevant to other ophthalmic implants, such as contact lenses or lacrimal punctal occluders (punctal plugs), which may act as favorable surfaces for bacterial colonization and infectious persistence [13,14].

In this context, the present study aimed to provide a comprehensive genotypic and phenotypic characterization of a series of bacterial isolates obtained from conjunctival samples of patients presenting for ophthalmological evaluation at the Ophthalmology Clinic in Timișoara. Furthermore, the study investigated the relationships between the carriage of antibiotic resistance genes and the corresponding phenotypic expression of antibiotic resistance, particularly in biofilm-forming isolates, in order to provide information relevant to the development of therapeutic strategies in ocular infections.

## 2. Results

### 2.1. Species Distribution

A total of 78 bacterial isolates were identified and analyzed, including 66 GPC (84.61%) and 12 GNB (15.38%). Gram-positive (GP) isolates were significantly more numerous than Gram-negative (GN) ones (*p* < 0.001). The GPC were assigned to the *Staphylococcus*, *Streptococcus*, and *Enterococcus* genera, with the distribution shown in Table 1. The coagulase-negative staphylococci (CoNS, *n* = 50) were the prevalent group, among which *S. epidermidis* was the most frequent (72%, *n* = 36), followed by *S. hominis* (14%, *n* = 7), *S. haemolyticus* (10%, *n* = 5), and *S. lugdunensis* (4%, *n* = 2). Considering the total number of isolates studied (*n* = 78), species distribution was as follows: *S. epidermidis* (46.15%, *n* = 36), *S. hominis* (8.97%, *n* = 7), *S. haemolyticus* (6.41%, *n* = 5), and *S. lugdunensis* (2.56%, *n* = 2). Among these, *S. epidermidis* had a significantly higher prevalence compared with *S. aureus* isolates (*p* < 0.001).

GNB comprised a wide range of species, but in a limited number of isolates. Accordingly, six isolates belonging to the *Enterobacterales* order were identified: *Escherichia coli* (*n* = 1), *Klebsiella pneumoniae* (*n* = 1), *Serratia marcescens* (*n* = 2), *Aeromonas hydrophila (n* = 1), and *Raoultella planticola* (*n* = 1). Additionally, five non-fermenting GNB were identified: *Acinetobacter baumannii* (*n* = 1), *Acinetobacter lwoffii* (*n* = 1), *P. aeruginosa* (*n* = 2), and *Ralstonia insidiosa* (*n* = 1), as well as one fastidious species, *Haemophilus influenzae* (*n* = 1).

### 2.2. Resistance Genes and Phenotypes

The identified resistance genes, AMR phenotypes, and biofilm-forming capacity are presented by species category.

#### 2.2.1. Gram-Positive Cocci

We analyzed the group of *S. aureus* and the group of CoNS species separately, considering their different levels of virulence and their known implications for ocular pathology. For the same reasons, we also presented *Streptococcus* group B and *Streptococcus viridans* group separately. Analysis of the 66 GPC showed that 75.75% (*n* = 50) harbored at least one of the resistance genes investigated. Among *S. aureus* isolates (*n* = 12), 83.33% (*n* = 10) carried resistance genes, while CoNS isolates (n = 50) showed a frequency of 78% (*n* = 39).

**Figure 1:**
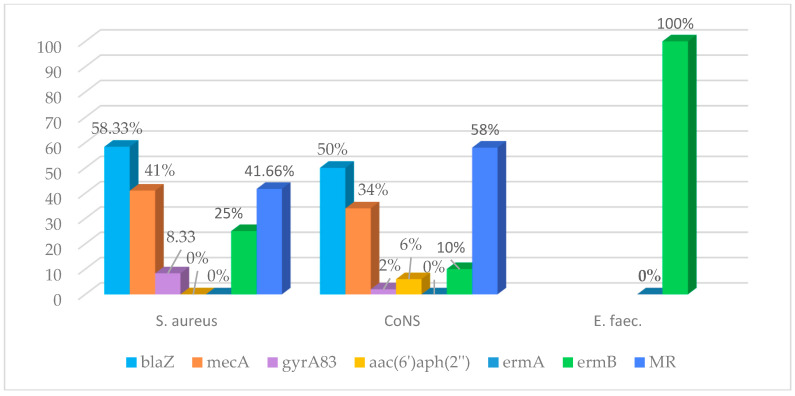
Resistance genes and MR phenotype in GPC. Legend: CoNS—coagulase-negative staphylococci; VGS—viridans group streptococci; *Str.* B—Group B *Streptococcus*; *E. faec.*—*Enterococcus faecalis*; *blaZ*—β lactamase gene; *mecA*—methicillin resistance gene encoding PBP2a; *gyrA83*—fluoroquinolone resistance gene; *aac(6′)/aph2(″)*—aminoglycoside resistance gene; *ermA, ermB*—macrolide-lincosamide-streptogramin B resistance genes; MR—methicillin resistance phenotype.

The presence of the *mecA* gene identified in *S. aureus* (41%) was phenotypically expressed by positive methicillin-resistant *S. aureus* (MRSA) screening and resistance to oxacillin. In the case of CoNS species, the proportion of isolates with a methicillin-resistant (MR) phenotype (58%) was significantly higher than that of isolates in which the *mecA* gene was detected (34%) (*p* = 0.026). A subset of *blaZ* and *mecA*-carrying isolates was associated, respectively, with resistance genes to macrolides and fluoroquinolones. Aminoglycoside resistance genes were identified only in CoNS, but their presence was sporadic and not statistically significant (*p* = 0.586). The incidence and distribution of resistance genes and MR phenotypes among GPC are shown in Figure 1, while the characteristics of each isolate are illustrated in Table 2.

The analysis of resistance phenotypes showed that multidrug-resistant Gram-positive cocci (MDR-GPC, *n* = 43) accounted for 65.15% of all GP isolates (*n* = 66). No isolates with an extensively drug-resistant (XDR) phenotype, corresponding to major antibiotic resistance, were recorded.

No statistically significant differences were observed between *S. aureus* and CoNS isolates regarding the carriage of *blaZ* (*p* = 0.750) and *mecA* (*p* = 0.739) genes, the expression of multidrug-resistant (MDR, *p* = 0.486) and MR phenotypes (*p* = 0.348), or moderate/strong biofilm-forming capacity (*p* = 0.108).

**Figure 2:**
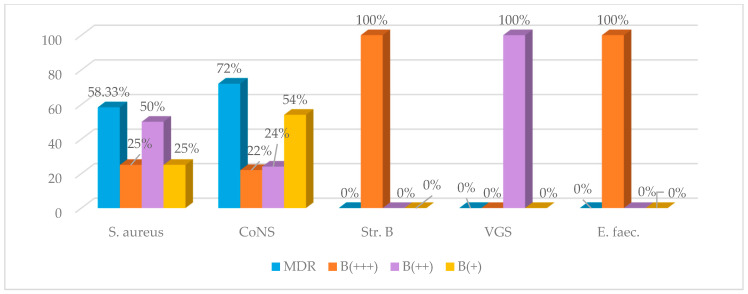
Distribution of MDR phenotypes and biofilm-forming capacity among GPC. Legend: CoNS—coagulase-negative staphylococci; VGS—viridans group streptococci; *Str.* B—Group B *Streptococcus*; *E. faec*.—*Enterococcus faecalis*; Biofilm formation: B+++ strong, B++ moderate, B+ weak producers.

The frequency of MDR-GPC isolates and their biofilm-forming capacity are illustrated in Figure 2.

As predicted, *S. aureus* and CoNS isolates carrying at least one resistance gene were classified as MDR in a significantly higher proportion, compared with those without resistance genes (*p* = 0.04) (Figure 2).

*S. epidermidis* isolates were MDR in 75% (12) of cases and formed moderate/strong biofilms in 41.66% (15) of isolates. *S. aureus* isolates were MDR in 58.33% of cases, while 75% exhibited moderate/strong biofilm formation. No statistically significant differences were observed between these profiles.

#### 2.2.2. Gram-Negative Bacilli

A total of 12 GNB were identified in the studied samples, including six isolates belonging to the *Enterobacterales* order, five non-fermenting GNB, and one *H. influenzae* isolate. Distribution of β-lactamase resistance genes and their biofilm-forming capacity are shown in Table 3.

Isolates carrying resistance genes accounted for approximately one third of all GNB (33.33%, *n* = 4), a significantly smaller proportion compared to that observed among GPC (*p* = 0.006).

The number of multidrug-resistant Gram-negative bacilli (MDR-GNB) was low (16.66%, *n* = 2), with a significantly smaller proportion (*p* = 0.002) than that observed among MDR-GPC (65.15%, *n* = 43). Two isolates were extended-spectrum β-lactamase (ESBL) producers (*E. coli* and *K. pneumoniae*), in which β-lactamase genes were identified. No XDR phenotype corresponding to extensive antibiotic resistance was detected.

### 2.3. Biofilm Formation

The biofilm-forming capacity among *S. aureus* was at least moderate in 75% (*n* = 9) of the total isolates, whereas among CoNS, it was observed in 46% (*n* = 23) of the isolates. No statistically significant differences regarding biofilm-forming capacity (*p* = 0.108) were found between *S. aureus* and CoNS species.

As concerns GPC, 22.72% (*n* = 15) were strong biofilm producers (+++), 31.81% (*n* = 21) showed moderate biofilm-forming capacity (++), and 45.45% (*n* = 30) were weak biofilm producers (+).

As regards GNB, 58.33% (*n* = 7) were strong biofilm producers (+++), 25% (*n* = 3) showed moderate biofilm-forming capacity (++), and 8.33% (*n* = 1) were weak biofilm producers (+).

GNB exhibited a significantly higher capacity to form strong biofilms compared with GPC (*p* = 0.03); however, no statistically significant difference was observed for isolates with moderate biofilm-forming capacity (*p* = 0.745). In contrast, a significantly higher number of GPC formed weak biofilms compared with GNB (*p* = 0.012).

The analysis of the association and the strength of the relationship between the presence of resistance genes and the biofilm-forming capacity (Table 4) indicated that in GNB the association was positive and of moderate strength but did not acquire statistical significance (φ = 0.386; *p* = 0.547). In the case of *S. aureus*, the association was positive, strong, and statistically significant (φ = 0.775; *p* = 0.027), whereas in CoNS, the association was positive, weak to moderate, without gaining statistical significance (φ = 0.326; *p* = 0.07).

### 2.4. Biofilm Resistance to Antibiotics

Differences between BMIC_50_ and MIC values were analyzed using the Wilcoxon Signed-Rank test.

In the case of *S. aureus*, analysis of susceptibility to AK indicated significantly higher resistance in biofilm-forming strains compared with planktonic strains. The mean ratio is shown in Table 5. LEV testing showed no statistically significant differences between the MIC values of planktonic and biofilm forms, although the mean ratio exceeded the value of 2.00. This finding indicates substantial variability in AMR among isolates, suggesting that AMR patterns are not consistent across all strains and the resistance to antibiotics is heterogeneous.

For *S. epidermidis* strains, AK testing demonstrated significantly higher resistance in biofilm forms compared with planktonic forms, with similar findings observed for LEV.

It should be noted that the mean MIC and BMIC_50_ values recorded for *S. epidermidis*, for both AK and LEV, were considerably higher than the corresponding mean values observed for *S. aureus* (Table 5).

Furthermore, in *S. epidermidis*, the higher values (z, *p*) obtained upon testing for both AK and LEV indicate that the resistance differences are highly consistent and present in most data pairs. In contrast, for *S. aureus*, the consistency of the effect is moderate to high but less uniform across pairs.

## 3. Discussion

AMR patterns, particularly among biofilm-forming bacteria, represent an emerging challenge in the management of ocular infections. Biofilms constitute an essential bacterial survival strategy and, in ocular surface infections, significantly contribute to bacterial persistence, treatment failure, and recurrent disease. In this study, bacterial isolates from conjunctival samples were classified into GP and GN groups, as GP bacteria are recognized as the predominant etiological agents of conjunctival infections in routine clinical practice. This approach facilitated a comparative evaluation of the biofilm-forming capacity, the presence of AMR-associated genes, and biofilm-related AMR among conjunctival bacterial isolates.

Recent studies conducted in different geographical regions indicate that GPC prevail among bacterial conjunctivitis, with reported frequencies ranging from 60% to over 80% of all isolates [15]. Accordingly, in investigations of ocular and periocular infections, GPC accounted for 56.5% of isolates, compared with 43.5% GNB, particularly CoNS (27.6%) and *S. aureus* (22.4%) [16]. A similar distribution has been reported in the pediatric population, where GP isolates are responsible for most acute bacterial conjunctivitis episodes [17]. These findings are consistent with data from our geographical region. In a previous study conducted in a tertiary care hospital in Timisoara, GPC accounted for 57.7% of conjunctival isolates, whereas GN pathogens were less frequent, represented by *Enterobacterales* (17.97%) and non-fermenting GNB (7.69%) [18]. By contrast, the prevalence of GNB, particularly *E. coli* and *P. aeruginosa*, has been reported in surgical ocular infections [19]. Taken as a whole, these findings support the approach of the present study, which primarily focuses on the analysis of AMR profiles and biofilm-forming capacity among conjunctival isolates.

Our results are comparable to those reported in the literature, with GPC being significantly more numerous than GNB. *Staphylococcus* spp. prevailed, with the most frequently isolated species being *S. epidermidis*. By contrast, *S. aureus* was identified at a significantly lower frequency.

A similar distribution has been reported in other studies, where GPC accounted for approximately 70.7% of all isolates from conjunctival samples, with *S. epidermidis* representing 36.2% and *S. aureus* 17.2% [20,21]. The presence of *S. epidermidis*, a common colonizer of the lacrimal canal and facial skin, highlights the ability of these opportunistic species to become an ocular pathogen under favorable conditions, regardless of systemic immunosuppression, trauma, or other pathological conditions. This capacity has also been documented in other studies, which reported the involvement of CoNS and *S. aureus* as frequent etiological agents of bacterial conjunctivitis across different age groups, confirming the role of *S. epidermidis* as an opportunistic ocular pathogen [22,23]. The pathogenic role of *S. epidermidis* is further supported by evidence from specific clinical settings, such as neonatal conjunctivitis. Bacteriological studies performed in hospitalized newborns have shown that GPC play a major etiological role, with *S. epidermidis* acting as the main pathogen in approximately 59.7% of cases, followed by *S. aureus* (21.7%) [24].

In the present study, *S. epidermidis* exhibited a MDR phenotype and a moderate to strong biofilm-forming capacity, statistically comparable to that observed for *S. aureus*, supporting the role of these species as active virulent opportunistic pathogens in conjunctival infections. Similar findings have been reported in other studies, which indicated the significantly higher prevalence of MDR and biofilm-forming *Staphylococcus* spp. in patients with vernal keratoconjunctivitis compared with healthy individuals [25]. The biofilm-forming capacity of *S. epidermidis* and *S. aureus* is also supported by other literature data, which showed that most bacterial isolates from contact lens wearers exhibited a moderate or strong biofilm formation based on phenotypic assessment, regardless of the presence of traditional biofilm-associated genes [26].

Analysis of *mecA* carriage in *Staphylococcus* spp. indicated higher prevalence among *S. aureus* isolates than among CoNS, although this difference was not statistically significant. Notably, MR phenotypes were identified in several *mecA*-negative CoNS isolates. This discrepancy may be explained by heteroresistance, technical limitations, or the involvement of alternative genetic determinants of MR, such as *mecC* genes.

There are numerous studies reporting this discrepancy in *Staphylococcus* isolates exhibiting MR phenotypes despite the absence of the mecA gene. Thus, non-*mecA* MR-CoNS strains have been described, in which MR was associated with the presence of the arcC metabolic gene [27]. In addition, some CoNS showing phenotypic resistance to oxacillin may lack the *mecA* gene and be classified as borderline oxacillin-resistant CoNS (BORCoNS). These strains exhibit phenotypic resistance despite the absence of classical mec determinants, which may explain discrepancies between methicillin resistance phenotypes and *mecA* detection in CoNS isolates [28].

In Maestre’s study, focused on MRSA keratitis isolates, a subset of strains classified as MRSA by conventional microbiological testing lacked detectable *mecA*, accounting for approximately 17.5% of cases, thereby supporting the concept that MR may occur through mechanisms other than the classical *mecA*-mediated pathway [29].

Another study reported that, despite the absence of *mecA* detection in phenotypically resistant isolates, alternative resistance determinants, including *mecC*, cannot be ruled out and should be explored in future molecular analyses [30]. Moreover, MR isolates lacking *mecA/mecC* have previously been attributed to mutations in native penicillin-binding proteins or to β-lactamase hyperproduction, characteristic of borderline oxacillin-resistant *S. aureus* (BORSA), which exhibit low-level oxacillin resistance without carrying the modified PBP2a [31]. Furthermore, another recent study has demonstrated that specific *blaZ* gene variants may confer a borderline oxacillin-resistant phenotype regardless of *mecA/mecC*, providing a potential molecular explanation for non-*mecA* MR isolates [32].

As regards GNB, a relatively low representation was observed in the present study, with only 12 identified isolates; however, this group was characterized by a high diversity of individually represented species belonging to the *Enterobacterales* order and the GN non-fermenting group. The majority of isolates showed moderate to strong biofilm-forming potential (over 90%), but more than 80% of them remained susceptible to the tested antibiotics. The increased biofilm-forming potential among certain GNB has also been reported in other studies, which showed that *P. aeruginosa* exhibited strong biofilm formation in 40% of isolates, whereas other GNB showed lower potential [33].

The relatively high antibiotic susceptibility observed in our study is consistent with long-term retrospective data on non-viral conjunctivitis, showing that GNB account for approximately 24% of isolates, with *Haemophilus* spp. as the prevalent and generally susceptible species [34]. This trend is further supported by international data indicating that common GN ocular pathogens, such as *P. aeruginosa* and *H. influenzae*, largely remain susceptible to tested antibiotics, whereas resistance is more frequently observed among GPC, particularly MR staphylococci [35].

In the present study, four isolates were carriers of β-lactamase resistance genes (TEM, SHV, CTX-M): *E. coli*, *K. pneumoniae*, *S. marcescens*, and *H. influenzae*.

The identified *H. influenzae* isolate demonstrated a TEM-producing phenotype, as indicated by resistance to ampicillin and susceptibility to amoxicillin/clavulanate. It was also susceptible to meropenem and fluoroquinolones (ciprofloxacin, levofloxacin). *H. influenzae* has been reported as an etiological agent of conjunctival infections, particularly in children, which may progress to severe systemic complications [36]. TEM-β-lactamase-producing strains associated with PBP3 modifications indicated significantly reduced susceptibility to β-lactam antibiotics [37].

The ESBL-producing *K. pneumoniae* isolate was the most important carrier of β-lactam resistance genes (TEM, SHV, CTX-M). It phenotypically expressed resistance to broad-spectrum cephalosporins (ceftazidime, cefepime), associated with resistance to aminoglycosides (gentamicin, tobramycin), sulfonamides (trimethoprim–sulfamethoxazole), and intermediate susceptibility to fluoroquinolones (ciprofloxacin, levofloxacin), displaying a MDR phenotype.

*E. coli* was the second ESBL-producing isolate, carrying TEM and CTX-M resistance genes. It exhibited a more complex MDR phenotype, with concomitant resistance to aminoglycosides (gentamicin) and fluoroquinolones (ciprofloxacin, levofloxacin).

*P. aeruginosa* is among the most frequently reported species associated with ocular infections and is commonly involved in conjunctivitis in contact lens wearers [38,39,40]. In the present study, two *P. aeruginosa* isolates with strong and moderate biofilm-forming capacity were identified. Both isolates remained susceptible to meropenem, amikacin, and tobramycin and showed dose-dependent susceptibility to ceftazidime, cefepime, ciprofloxacin, and levofloxacin. Notably, the strongly biofilm-forming *P. aeruginosa* strain was isolated from a permanent contact lens wearer presenting with clinical signs of conjunctival infection. This strain did not exhibit an MDR phenotype.

In the present study, a *Ralstonia insidiosa* isolate with strong biofilm-forming capacity was also identified, showing resistance to third-generation cephalosporins in the absence of detectable resistance genes. Another case of conjunctivitis caused by *Ralstonia pickettii* in an immunocompetent contact lens wearer has also been reported in the literature [41].

Analysis of resistance gene carriage in the present study showed that the proportion of GPC harboring antibiotic resistance genes was significantly higher than that of GNB. Moreover, the genes identified in GNB isolates were exclusively β-lactamase–type, although the screening targeted a broad spectrum of resistance determinants. Both GP and GN isolates exhibited an MDR phenotype; however, MDR-GPC were significantly more numerous than MDR-GNB, which may be explained by the expanded genetic arsenal of GP isolates. As regards the biofilm-forming capacity, however, GNB showed significantly higher potential compared to GPC.

The relationship between resistance gene carriage and biofilm-forming capacity in the GP and GN groups showed a positive association; this association was moderate in GNB, but strong and statistically significant in *S. aureus* isolates, suggesting that the latter combine antibiotic resistance genes with the genetic and biochemical mechanisms involved in bacterial biofilm formation.

In the present study, biofilm susceptibility testing was performed using AK and LEV as representative agents from two antibiotic classes commonly used in ophthalmic practice. The selection of these antibiotics was intended to explore the relationship between AMR genes, phenotypic resistance, and biofilm-associated antimicrobial tolerance in conjunctival isolates rather than to provide a comprehensive AMR surveillance analysis. Nevertheless, the use of a broader range of antibiotics, including newer ophthalmic fluoroquinolones, could provide a more detailed resistance profile and should be considered for future investigations.

Microorganisms within biofilms exhibit increased AMR, with *S. epidermidis* showing more prominent and uniform increases in MIC and BMIC_50_ values than *S. aureus*. Biofilm-associated resistance can significantly reduce the effectiveness of antimicrobial therapy, as susceptibility interpretations based on planktonic MIC values may underestimate the concentrations required to eradicate biofilm-embedded bacteria. Additionally, biofilm persistence may contribute to relapse once antibiotic therapy is discontinued.

The results of the present study may be influenced by the relatively small number of isolates, collected from a single center. In addition, no data were available regarding prior administration of antibacterial agents before sampling, which would have allowed correlation with the resistance phenotype. Moreover, testing a limited number of antibiotics from each class could not provide a complete characterization of resistance phenotypes to fluoroquinolones and aminoglycosides.

Despite these limitations, this study provides valuable data on the genotypic characterization of AMR and the biofilm-forming capacity of bacterial isolates from conjunctival samples. To our knowledge, this is the first study of this type conducted in the western region of our country. Overall, these findings underscore the clinical relevance of resistance gene detection and biofilm characterization in selected clinical contexts, such as the prevention of persistent or recurrent conjunctival infections and the management of complex perioperative settings, including preoperative assessment prior to cataract surgery. In this framework, extended microbiological characterization of conjunctival isolates in high-risk patients may contribute to optimized perioperative antimicrobial strategies and improved infection prevention.

## 4. Materials and Methods

We conducted a prospective, single-center, cross-sectional, observational study between November 2024 and May 2025, analyzing bacterial isolates obtained from conjunctival samples of patients examined in the Ophthalmology Department of the Municipal Hospital of Timișoara, Romania, a tertiary ophthalmology center affiliated with the Victor Babeș University of Medicine and Pharmacy of Timișoara and serving the western region of Romania.

The study aimed to identify antibiotic resistance genes at the molecular level, to characterize resistance phenotypes based on antibiotic susceptibility testing, to evaluate biofilm-forming capacity, and to analyze the relationship between genetic carriage and biofilm resistance to selected antibiotics.

Inclusion criteria consisted of the presence of clinical signs indicating unilateral or bilateral ocular inflammation or infection, such as conjunctival hyperemia, ocular pain, foreign body sensation or discomfort, and mucous, mucopurulent, or purulent ocular discharge, with or without associated eyelid edema. Patients were recruited from both outpatient and inpatient ophthalmology services.

Exclusion criteria included inadequate biological specimens or insufficient conjunctival secretions for microbiological analysis, cases with non-infectious ocular conditions of allergic origin, patients with a history of recent ocular surgery or chronic ocular surface disease, and immunocompromised patients.

Conjunctival secretion sampling was performed by an ophthalmologist using sterile swabs, avoiding contact with the eyelid margins and eyelashes to prevent contamination. The samples were then transported in Amies transport medium to the Municipal Hospital’s clinical laboratory for bacteriological diagnosis. The clinically significant isolates were cryopreserved at −78 °C in brain heart infusion broth (BHI; Thermo Fisher Scientific, Oxoid Deutschland GmbH, Wesel, Germany) using an UNYCRO deep freezer (−85 °C; UniEquipP, Munich, Germany). Molecular analyses and bacterial biofilm-forming capacity assays were performed after the complete sample set had been established.

Written informed consent was obtained from all patients (or their legal representatives, when applicable) for conjunctival sampling and the use of their clinical and paraclinical data for research purposes. All data were anonymized prior to analysis to ensure patient confidentiality.

### 4.1. Resistance Phenotype Analysis

Identification of bacterial isolates, antimicrobial susceptibility testing (AST), and determination of MIC were performed in accordance with the protocols of the Microbiology Laboratory, using E-tests and the automated VITEK^®^ 2 Compact system (bioMérieux, Marcy-l'Étoile, France), as well as Matrix-Assisted Laser Desorption/Ionization Time-of-Flight mass spectrometry (MALDI Biotyper, Bruker, Bremen, Germany). Interpretation of antimicrobial susceptibility results was carried out according to the EUCAST 2024 guidelines [42]. 

The strains included in the study were characterized based on their resistance phenotypes, defined as follows: (1) MRSA, defined as *S. aureus* isolates with an oxacillin MIC ≥ 4 mg/L; (2) MDR bacteria, defined as resistance to at least one agent in three or more antimicrobial classes active against the respective species [43]; (3) XDR bacteria, defined as resistance to at least one agent in all but one or two antimicrobial classes [43]; (4) ESBL-producing GNB, identified by resistance to third- or fourth-generation cephalosporins and monobactams and confirmed by synergy testing; and (5) carbapenem-resistant GNB, defined as an MIC ≥ 4 mg/L for imipenem or meropenem (≥8 mg/L for non-fermenting GNB) [42].

### 4.2. PCR Detection of Antibiotic Resistance Genes

GP isolates were screened for the presence of genes associated with β-lactam, methicillin, macrolide, fluoroquinolone, and aminoglycoside resistance, whereas GN were investigated for genes conferring resistance to β-lactams, carbapenems, and aminoglycosides. The primers used in this study are listed in Table 6.

Microbial DNA was extracted from overnight cultures of the isolates using the PureLink™ Microbiome Purification Kit (Thermo Fisher Scientific, Waltham, MA, USA), according to the manufacturer’s instructions. The qPCR reactions employing hydrolysis probes (TaqMan-type assays) were performed in a final reaction volume of 20 µL, containing 10 µL of 2× master mix, specific primers, hydrolysis probe, and 3 µL of purified DNA template. Amplification and fluorescence detection were carried out using the Azure Cielo Real-Time PCR System (Azure Biosystems, Inc., Dublin, CA, USA), in accordance with the manufacturer’s recommendations for the master mix.

### 4.3. Determination of Biofilm-Forming Capacity

The biofilm-forming capacity was assessed using the microtiter plate assay, according to the protocol described by Stepanović et al. [44]. Sterile, flat-bottom, 96-well polystyrene microtiter plates were used.

The samples were inoculated onto standard culture media (from Thermo Fisher Scientific, Oxoid Deutschland GmbH, Wesel, Germany): blood agar, mannitol salt agar, MacConkey agar, chromogenic agar, or chocolate agar, as appropriate, and incubated at 37 °C for 24 h. Isolates grown on agar media were then inoculated into 2 mL of brain heart infusion broth (BHI) supplemented with 1% glucose to enhance biofilm formation and incubated at 37 °C for 24 h. The BHI medium had the following composition (g/L): brain infusion solids 12.5, beef heart infusion solids 5.0, proteose peptone 10.0, glucose 2.0, sodium chloride 5.0, and disodium phosphate 2.5, with a final pH of 7.4 ± 0.2. Glucose-enriched BHI broth was selected for the study of biofilms because it is a nutrient-rich medium and is used, according to protocols, for biofilm formation assays [45,46,47].

**Figure 3:**
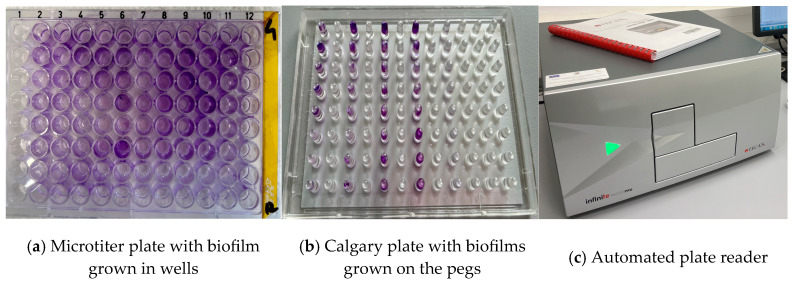
Biofilm detection.

In the subsequent step, the resulting cultures were diluted 1:100 in fresh medium, and 200 µL of the diluted suspension was inoculated into each well of the microtiter plates. The plates were incubated at 37 °C for 24 h to allow biofilm formation. After incubation, the contents of the wells were removed, and the plates were washed three times with 200 µL of phosphate-buffered saline (PBS; pH 7.2). The plates were then incubated for 1 h at 60 °C, followed by staining with 1% crystal violet. Excess stain was removed by rinsing the plates three times with deionized water, after which the bound dye was solubilized using 30% acetic acid. (Figure 3).

The optical density (OD) of the stained adherent biofilm was measured at a wavelength of 570 nm using an automated micro-ELISA plate reader. For quality control (QC), the reference strains *Pseudomonas aeruginosa* ATCC 27853 and *Staphylococcus aureus* ATCC 29213 were used [48]. Each isolate was tested in three wells. Uninoculated wells containing only broth served as the negative control. Biofilm production was interpreted according to the criteria proposed by Kırmusaoğlu et al. [49].

Wells corresponding to isolates whose optical density (OD) values exceeded those of the negative control were considered positive for biofilm production. The cut-off optical density (ODc) was calculated using the following formula: ODc = mean OD of the negative control + (3 × standard deviation [SD] of the negative control). Subsequently, the adjusted OD value for each isolate was calculated as follows: OD isolate = mean OD of the isolate − ODc.

Based on their optical density values, the results were classified into four categories, as follows: (1) strong biofilm producers (OD > 4 × ODc); (2) moderate biofilm producers (2 × ODc < OD ≤ 4 × ODc); (3) weak biofilm producers (ODc < OD ≤ 2 × ODc); (4) non-biofilm-producing strains (OD ≤ ODc). Accordingly, strong (+++), moderate (++), and weak (+) biofilm-forming strains, as well as non-biofilm-forming strains, were identified in accordance with the criteria suggested by Kırmusaoğlu et al. [49].

### 4.4. Antibiotic Susceptibility Testing of Biofilms

#### 4.4.1. Preparation of Antibiotic Stock Solutions

We used LEV (Levofloxacin L001-1G; Toku-E, Centennial, CO, USA) and AK (Amikacin A002-1 g; Toku-E, Centennial, CO, USA) for both standard antimicrobial susceptibility testing of bacterial isolates and biofilm susceptibility assays. Stock solutions were prepared at a concentration of 10 mg/mL and stored at −20 °C until use. Serial dilutions ranging from 0.125 to 128 µg/mL were subsequently prepared from the stock solutions.

#### 4.4.2. Biofilm Susceptibility Testing to LEV and AK

Biofilm susceptibility testing to antibiotics was performed using Calgary plates (MBEC Assay^®^ Biofilm Inoculator with 96-Well Base, catalog no. 19113, Innovotech, Edmonton, AB, Canada), according to a modified protocol described by Pandelis et al. [50].

For this purpose, 100 µL of a 1:100 dilution of an overnight bacterial culture was added to 100 µL of BHI supplemented with 1% glucose in each well of a 96-well Calgary plate. The plate lid was placed to allow complete immersion of the pegs in the bacterial suspensions. Plates were incubated at 37 °C for 48 h under gentle agitation to allow biofilm formation on the surface of the pegs.

After incubation, the peg lids were subjected to two consecutive washing steps to remove planktonic bacteria by sequential transfer of the pegs into two 96-well plates containing PBS. Following the second wash, the lids were transferred to another 96-well plate containing serial dilutions of meropenem and amikacin, with each concentration tested in triplicate, along with antibiotic-free control wells.

The plates were incubated at 37 °C for an additional 48 h under gentle agitation to allow biofilm exposure to the antibiotics. Subsequently, the peg lids were washed twice with PBS to remove residual antibiotics and then transferred to a recovery medium plate. Detachment of the biofilm from the pegs was achieved by vortexing, allowing the release of biofilm-embedded bacteria into the wells.

After removal of the peg lids, they were replaced with sterile standard lids, and biofilm evaluation was performed by crystal violet staining followed by optical density measurement. For this study, the MIC_50_ value was determined and defined as the minimum antibiotic concentration that inhibited 50% of biofilm-embedded bacteria compared with the growth observed in the positive control wells. Biofilm formation was confirmed by bacterial growth observed in the antibiotic-free positive control wells, in accordance with previously described protocols [48,50,51].

### 4.5. Statistical Analysis

The database was analyzed using IBM SPSS Statistics 20 (SPSS Inc., Chicago, IL, USA). Nominal variables were expressed as values and percentages, and the chi-square (χ^2^) test was used for comparison, with application of Fisher’s exact test. To assess the strength of the association between the presence of resistance genes and the biofilm-forming capacity, Cramer’s V coefficient was used. The threshold for statistical significance was set at ≤0.05, and all tests were two-tailed.

The normality of MIC difference distributions between planktonic and biofilm-associated isolates was assessed using the Shapiro–Wilk test. As the distribution deviated significantly from normality (*p* < 0.05), MIC differences were analyzed using the non-parametric Wilcoxon signed-rank test for paired samples.

## 5. Conclusions

The identified GPC were significantly more numerous than GNB, with MDR-GPC considerably more frequent than MDR-GNB. GNB showed, however, significantly higher biofilm-forming potential compared with GPC, but the relatively small number of GN isolates may restrict the statistical robustness of inter-group comparisons and the generalizability of the findings. *S. aureus* showed a strong, positive, and statistically significant association between the carriage of resistance genes and the genetic and biochemical mechanisms involved in bacterial biofilm formation. Additionally, biofilm-associated isolates showed increased AMR compared with their planktonic forms, highlighting the clinical relevance of biofilm-related resistance. The combined evaluation of AMR profiles and biofilm-forming capacity provides valuable information for optimizing empirical therapy and preventing persistent or recurrent conjunctival infections. These findings emphasize the importance of continuous surveillance of AMR and biofilm formation among conjunctival isolates in order to regulate therapeutic strategies and limit the emergence of difficult-to-treat ocular infections.

## Figures and Tables

**Table 1:** Distribution of GPC and GNB isolates in the study group.

Species	GPC 84.61 (66)					GNB	Total
	*S. aureus*	CoNS	*E. faecalis*	*Str.* B	VGS		
% (*n*)	15.38 (12)	64.10 (50)	1.28 (1)	1.28 (1)	2.56 (2)	15.38 (12)	100% (78)

Legend: GPC = Gram-positive cocci; GNB = Gram-negative bacilli; CoNS = coagulase-negative staphylococci; *Str.* B = group B *Streptococcus*; VGS = viridans group streptococci. Values are expressed as percentage (*n*, number of isolates).

**Table 2:** Gram-positive cocci carrying resistance genes—resistance phenotypes, biofilm formation.

Species	*blaZ*	*mecA*	*gyrA83*	*aac(6′)/* *aph(2″)*	*ermA*	*ermB*	MR	MDR	Biofilm		
									(+++)	(++)	(+)
*SA 1*	■	□	□	□	□	□	□	□	-	-	■
*SA 2*	■	□	□	□	□	□	□	□	-	■	-
*SA 3*	□	■	□	□	□	□	■	□	■	-	-
*SA 4*	■	□	■	□	□	■	□	□	-	-	■
*SA 5*	■	□	□	□	□	□	□	□	-	■	
*SA 6*	□	■	□	□	□	■	■	□	-	■	
*SA 7*	□	■	□	□	□	□	■	□	-		■
*SA 8*	■	□	□	□	□	□	□	□	-	■	
*SA 9*	■	■	□	□	□	□	■	□	-	■	
*SA 10*	■	■	□	□	□	■	■	□	-	-	
*S. epid. 1*	■	■	□	□	□	□	■	■	-	-	■
*S. epid. 2*	■	□	□	□	□	□	□	■	-	-	■
*S. epid. 3*	■	□	□	□	□	□	□	□	-	-	■
*S. epid. 4*	■	■	■	□	□	□	■	■	-	-	■
*S. epid. 5*	■	□	□	□	□	□	■	■	-	-	■
*S. epid. 6*	■	□	□	□	□	□	■	■	■	-	-
*S. epid. 7*	□	■	□	□	□	□	■	■	-	■	-
*S. epid. 8*	■	■	□	□	□	□	■	■	-	■	-
*S. epid. 9*	■	□	□	□	□	■	■	□	-	-	■
*S. epid. 10*	■	□	□	□	□	□	□	□	-	■	
*S. epid. 11*	■	□	□	□	□	□	□	□	-	-	■
*S. epid. 12*	■	□	□	□	□	■	■	■	-	■	-
*S. epid. 13*	■	□	□	□	□	■	■	■	-	-	■
*S. epid. 14*	■	□	□	□	□	■	■	■	-	-	■
*S. epid. 15*	□	□	□	□	□	■	■	■	-	-	■
*S. epid. 16*	■	□	□	□	□	□	□	□	-	-	■
*S. epid. 17*	■	□	□	□	□	□	■	□	-	-	■
*S. epid. 18*	■	□	□	□	□	□	□	□	-	-	■
*S. epid. 19*	□	□	□	■	□	□	■	□	■	-	-
*S. epid. 20*	□	■	□	□	□	■	■	■	-	■	-
*S. epid. 21*	□	■	□	□	□	■	■	■	-	■	-
*S. epid. 22*	□	■	□	□	□	■	■	■	-	-	■
*S. epid. 23*	□	■	□	■	□	■	■	■	-	-	■
*S. epid. 24*	□	■	□	□	□	■	■	■	■	-	-
*S. epid. 25*	■	■	□	□	□	■	■	■	■	-	-
*S. epid. 26*	□	■	□	□	□	■	■	■	■	-	-
*S. epid. 27*	■	■	□	□	□	■	■	■	■	-	-
*S. epid. 28*	□	■	□	□	□	■	■	■		■	-
*S. epid. 29*	□	■	□	□	□	■	■	■	■	-	-
*S. hom. 1*	■	□	□	□	□	■	■	■	■	-	-
*S. hom. 2*	■	□	□	□	□	□	■	□	-	-	■
*S. hom. 3*	□	■	□	■	□	□	■	■	■	-	-
*S. hom. 4*	□	■	□	■	□	□	■	■	-	■	-
*S. hom. 5*	■	■	□	■	□	□	■	■	-	■	-
*S. haem. 1*	■	□	□	□	□	□	□	□	■	-	-
*S. haem. 2*	■	□	□	□	□	□	■	■	■	-	-
*S. haem. 3*	■	□	□	□	□	□	■	■	-	■	-
*S. lund. 1*	■	□	□	□	□	□	□	□	-	-	■
*S. lund. 2*	□	□	□	□	□	■	□	□	-	-	■
*Str. mitis*	/	/	/	/	□	□	□	□	-	■	-
*Str.* gr. B	/	/	/	/	□	□	□	□	■	-	-
*Str. oralis*	/	/	/	/	□	□	□	□	-	■	-
*E. faecalis*	/	/	/	/	□	■	□	□	■	-	-

Legend: SA—*Staphylococcus aureus*; *S. epid.*—*Staphylococcus epidermidis*; *S. hom.*—*Staphylococcus hominis*; *S. haem.*—*Staphylococcus haemolyticus*; *S. lund.*—*Staphylococcus lugdunensis*; *Str.* gr. B—Group B *Streptococcus*; (/)—not applicable; MR—methicillin-resistant; MDR—multidrug-resistant phenotype; Biofilm formation: (+++) strong, (++) moderate, (+) weak producers. □—absent; ■—present; (-) absent, for each isolate there can be only one biofilm variant.

**Table 3:** Gram-negative bacilli carrying resistance genes—resistance phenotypes, biofilm formation.

Species	No. of Strains	Identified Resistance Genes	ESBL	MDR	Biofilm		
					(+++)	(++)	(+)
*Acinetobacter baumannii*	1	□	□	□	-	-	■
*Acinetobacter lwoffii*	1	□	□	□	■	-	-
*Aeromonas hydrophila*	1	□	□	□	-	■	-
*Escherichia coli*	1	*bla*TEM, *bla*CTX-M	■	■	-	■	-
*Haemophilus influenzae*	1	*bla*TEM	□	□	■	-	-
*Klebsiella pneumoniae*	1	*bla*TEM, *bla*SHV, *bla*CTX-M	■	■	■	-	-
*Pseudomonas aeruginosa*	2	□	□	□	■	■	-
*Ralstonia insidiosa*	1	□	□	□	■	-	-
*Raoultella planticola*	1	□	□	□	■	-	-
*Serratia marcescens*	2	*bla*TEM (1 strain)	□	□	■	■	-

Legend: ESBL—extended-spectrum β-lactamase; MDR—multidrug-resistant phenotype; □—absent; ■—present; (-) absent, for each isolate there can be only one biofilm variant; *bla*TEM = TEM-type β-lactamase gene; *bla*SHV = SHV-type β-lactamase gene; *bla*CTX-M = CTX-M-type β-lactamase gene; Biofilm formation: (+++) strong, (++) moderate, (+) weak producers.

**Table 4:** Association between the presence of resistance genes and biofilm-forming capacity in GPC and GNB—association strength and statistical significance.

Association Analyzed	Fisher’s Exact Test (*p*)	Cramer’s V	Interpretation
GNB—resistance genes/biofilm-forming capacity	*p* = 0.547	V = 0.386	moderate, positive association, not statistically significant
SA—resistance genes/biofilm-forming capacity	*p* = 0.027	V = 0.775	strong and positive association, statistically significant
CoNS—resistance genes/biofilm-forming capacity	*p* = 0.07	V = 0.326	weak to moderate, positive association, not statistically significant

Legend: GPC—Gram-positive cocci; GNB—Gram-negative bacilli; SA—*Staphylococcus aureus*; CoNS—coagulase-negative staphylococci.

**Table 5:** Differences in resistance to AK and LEV between planktonic and biofilm strains of *S. aureus* and *S. epidermidis*.

Study Association	z	*p*	Mean Ratio	Interpretation
*S. aureus*—AK BMIC_50_/MIC	2.980	0.003	2.416/0.791 = 3.054	Statistically significant difference; large effect size
*S. aureus*—LEV BMIC_50_/MIC	1.832	0.067	2.833/1.197 = 2.366	Non-significant difference; medium-to-large effect size
*S. epidermidis*—AK BMIC_50_/MIC	4.466	<0.001	8.472/3.722 = 2.276	Statistically significant difference; large effect size
*S. epidermidis*—LEV BMIC_50_/MIC	4.571	<0.001	5.416/3.163 = 1.712	Statistically significant difference; large effect size

Legend: AK—amikacin, LEV—levofloxacin.

**Table 6:** Primer sets used for detection of resistance genes in GN and GP isolates.

GN—Primer Sets	
TEM	F: TTGCACAACATGGGGGATC, R: AGCTAGAGTAAGTAGTTCGCCAGTTAATAGTT
SHV	F: CGATAACAGCGCCGCC, R: TTCCCAGCGGTCAAGGC
CTX-M-1 gr.	F: CTGGGTGTGGCATTGATTAACA, R: CTCGCTGATTTAACAGATTCGGTT
OXA-48	F: TGTTTATCAAGAATTTGCCCGC, R: TTCGGTCAGCATGGCTTGT
OXA-23	F: GACACTAGGAGAAGCCATGAAG, R: CAGCATTACCGAAACCAATAC
OXA-24	F: GATGACCTTGCACATAACCG, R: CAGTCAACCAACCTACCTGTG
NDM	F: ATTAGCCGCTGCATTGAT, R: CATGTCGAGATAGGAAGTG
VIM	F: GAGTTGCTTTTGATTGATACAG, R: TCGATGAGAGTCCTTCTAGA
aac(6′)-Ib	F: AACTTGCGAGCGATCCGA, R: TGGCGTGTTTGAACCATGTAC
**GP—Primer sets**	
*mecA*	F: CAATGCCAAAATCTCAGGTAAAGTG, R: AACCATCGTTACGGATTGCTTC
*blaZ*	F: GCTTTAAAAGAACTTATTGAGGCTTCA, R: CCACCGATYTCKTTTATAATTT
gyrA83	F: TACCATCCCCATGGTGACTC, R: GCCATGCGGACAATCGTGTC
ermA	F: AAACCGGTAAACCCCTCTGA, R: TTCGCCATTTGGGGAGACT
ermB	F: CATTTAACGACGAAACTGGC, R: GGAACATCTGTGGTATGGCG
aac(6′)/aph(2″)	F: TACAGAGCCTTGGGAAGATG, R: CATTTGTGGCATTATCATCATATC

Legend: GN = Gram-negative; GP = Gram-positive; F = Forward primer; R = Reverse primer; Forward and reverse primer sequences are shown in the 5′–3′ direction.

## Data Availability

The data supporting the findings of this study are available from the corresponding author upon reasonable request.

## Abbreviations

The following abbreviations are used in this manuscript:

AMR	Antimicrobial resistance
AST	Antimicrobial susceptibility testing
MIC	Minimum inhibitory concentration
AK	Amikacin
LEV	Levofloxacin
GPC	Gram-positive Cocci
GNB	Gram-negative bacilli
GP	Gram-positive
GN	Gram-negative
CoNS	Coagulase-negative staphylococci
MRSA	Methicillin-resistant *Staphylococcus aureus*
MR	Methicillin-resistant
MDR-GPC	Multidrug-resistant Gram-positive cocci
XDR	Extensively drug-resistant
MDR	Multidrug-resistant
MDR-GNB	Multidrug-resistant Gram-negative bacilli
ESBL	Extended-spectrum β-Lactamase
MR-CoNS	Methicillin-resistant Coagulase-negative staphylococci
BHI	Brain heart infusion
PBS	Phosphate-buffered saline
OD	Optical density
QC	Quality control
ODc	Cut-off optical density
SD	Standard deviation
